# Application of eculizumab, a terminal complement inhibitor, in the management of atypical hemolytic uremic syndrome in a 14-month-old Chinese pediatric patient: a case report

**DOI:** 10.3389/fped.2024.1404725

**Published:** 2024-07-31

**Authors:** Xin Wei, Xinzhu Liu, Yingying Yu, Wei Xie, Wentao Luo, Ye Tu, Shuhong Bu, Guimei Guo

**Affiliations:** ^1^ Department of Clinical Pharmacy, Xinhua Hospital, Shanghai Jiaotong University School of Medicine, Shanghai, China; ^2^ Department of Pediatric Intensive Care Unit, Xinhua Hospital, Shanghai Jiao Tong University School of Medicine, Shanghai, China; ^3^ Department of Pharmacy, Shanghai East Hospital, School of Medicine, Tongji University, Shanghai, China; ^4^ Department of Pediatric Nephrology, Rheumatology and Immunology, Xinhua Hospital, Shanghai Jiaotong University School of Medicine, Shanghai, China

**Keywords:** eculizumab, complement, aHUS, thrombotic microangiopathy, pediatric

## Abstract

Eculizumab, a recombined humanized monoclonal antibody which possesses high affinity for the complement protein C5, is a therapeutic agent utilized in the treatment of atypical hemolytic uremic syndrome (aHUS) by inhibiting the terminal complement complex C5b-9. In a pediatric patient with aHUS of 14 months, the administration of eculizumab therapy was accompanied by the inclusion of meningococcal vaccine as part of the national immunization program. Notably, no other antibiotics were administered prior to or during the course of eculizumab treatment. Moreover, there were no occurrences of infusion reactions or meningococcal infections observed throughout the course of treatment. Due to the presence of anti-factor H antibodies and insufficient recovery, glucocorticoids and eculizumab were administered at week 0 and week 1, followed by the initiation of mycophenolate mofetil (MMF) at a dosage of 250 mg (approximately 548 mg/m^2^) per day starting from Day 10. Due to the recovered of complement antibody after 8 doses of eculizumab, the therapeutic interval was extended from once every 3 weeks to once a month since 9th administration. We experienced and successfully treated a rare case of aHUS with eculizumab in a 14-month-old Chinese pediatric patient.

## 1 Introduction

Development of eculizumab In the early 1990s, investigators interested in developing complement inhibitors for therapy complement-mediated disease chose C5 as a target because of its central position within the cascade (1, 2). Inhibition of C5 not only prevents formation of terminal complement complex C5b-9 but also inhibits generation of C5a, the potent proinflammatory peptide that is released when C5 is cleaved by a C5 convertase. Another hypothetical advantage of targeting C5 is that immunoprotective (e.g., C3b-mediated opsonisation) and immunomodulatory (e.g., immune-complex solubilisation and clearance) processes that are effected by upstream constituents of the complement system remain unperturbed. In early clinical trials, eculizumab was utilized to treat patients with systemic lupus erythematosus (3), rheumatoid arthritis, and membranous nephropathy (4). Eculizumab received FDA approval for the treatment of paroxysmal nocturnal hemoglobinuria (PNH) in 2007 (1) and atypical hemolytic uremic syndrome (aHUS) (5).

aHUS is a rare, genetic, life-threatening systemic disease (5). Actually, it is a form of thrombotic microangiopathy (TMA) caused by the abnormal activation of complement (5). Before the application of eculizumab in the treatment of aHUS, plasma exchange (PE) was the first-line treatment. While PE has been reported to improve hematological parameters, long-term outcomes were often poor (6, 7) (PMID: 20595690, PMID: 23307876). Thus, a US guideline (8) (PMID: 27497856) recommends PE for “complement-mediated TMA” aHUS, but this approach is not supported by another consensus opinion (9) (PMID: 29419916). Moreover, the limited availability of blood products in clinical practice presents a therapeutic dilemma. Terminal complement inhibitor Eculizumab, was approved as a therapeutic drug and has transformed outcomes in aHUS patients (5). Eculizumab can promptly alleviate acute symptoms, reduce the use of blood products, and in this case, the patient did not experience any severe adverse reactions.

Simultaneously, this advancement has sparked numerous contentious issues, primarily centering on the ideal treatment duration (10). The potential risks of infection and elevated treatment expenses associated with eculizumab administration are weighed against the possibilities of aHUS relapse and exacerbated renal impairment upon discontinuation of eculizumab (10).

Nevertheless, the decision regarding the indefinite continuation of anticomplement therapy remains undetermined. Earlier research has indicated that the discontinuation of eculizumab in aHUS patients, guided by complement genetics, is both rational and safe (10). Herein, we present our recent experience with a 14-month-old pediatric patient diagnosed with aHUS and treated with Eculizumab.

## 2 Case report

This 1-year-old female (born on March 4th, 2022) pediatric patient presented to Xinhua Hospital affiliated with Shanghai Jiao Tong University School of Medicine on June 3rd, 2023 (day-7) with a history of “repeated vomiting and diarrhea for 3 days”. Two days prior to admission (day -8), the patient had one episode of dark brown paste-like stool after consuming cold drinks, which had no foul odor, blood, or pus, and was not associated with significant abdominal pain or bloating. On the day prior to admission (day -7), the patient started vomiting, with a total of 8–9 episodes. Initially, the vomitus consisted of gastric contents, but gradually changed to clear water-like fluid and eventually became dark green in color. The vomiting was accompanied by diarrhea, with 2 bowel movements per day. The stool initially had a paste-like consistency but later became watery. Throughout the course of illness, the patient did not have fever, cough, sputum production, or discomfort during urination. The outpatient blood routine examination conducted on the same day as the visit showed a C-reactive protein (CRP) level of less than 1 mg/L, a white blood cell count (WBC) of 9.03 × 10^9^/L, a hemoglobin (Hb) level of 119 g/L, and a platelet count (PLT) of 113 × 10^9^/L. The patient was prescribed cefprozil for suspension (0.13 g po bid) for oral administration, but there was no significant improvement after 1 day (day -6), leading to the diagnosis of “acute gastroenteritis” and subsequent hospitalization. The patient had an unremarkable medical history and received regular vaccinations according to the national immunization program. Her parents were healthy, denied consanguinity, denied any history of inherited metabolic diseases or any history of renal diseases. The patient weighed 9 kg and had a height of 77 cm, with a body surface area of 0.44 m^2^. On admission, vital signs were as follows: body temperature 36.2°C, respiratory rate 23 breaths/min, pulse rate 106 beats/min, blood pressure 117/85 mmHg. The girl was alert but slightly irritable, with a slightly decreased level of consciousness. Physical examination revealed a supple neck and no yellowing of the skin or mucous membranes. There were no rashes or sunken eye sockets. The cardiovascular, respiratory, abdominal, and neurological examinations were unremarkable, and there was normal muscle tone in all four limbs.

On the first day of hospitalization (Day -5), the patient experienced recurrent seizures and diarrhea, along with mild edema in the eyelids and throughout the body. Bilateral Babinski signs were positive, raising suspicion of viral encephalitis. The patient was treated with methylprednisolone (9 mg ivgtt q12h, from day -5 to week 2) for immunosuppression, and supportive therapy with blood products (human albumin, human immunoglobulin pH4, and packed red blood cells). After 1 day of treatment, the seizures resolved, and the results of targeted next-generation sequencing for pathogens in the cerebrospinal fluid and comprehensive virus antibody testing were inconclusive for viral encephalitis.

On the third day of admission (Day -3), the direct antiglobulin test (Coombs' test) and autoantibodies were negative. Hematological assessment revealed anemia and thrombocytopenia, evidenced by reduced Hb at 82 g/L (<100 g/L) and PLT at 38 × 10^9^/L (<150 × 10^9^/L). Additionally, an elevated reticulocyte (Ret) of 2.44% (normal range: 0.5%∼1.5%) was observed. Serum creatinine (Cr) levels were found to be elevated at 90 μmol/L, surpassing the upper normal limit of 33 μmol/L for healthy children of the same age and sex. Collectively, these findings are suggesting a diagnosis of TMA with features of hemolytic anemia, thrombocytopenia, and renal dysfunction.

**Figure 1:**
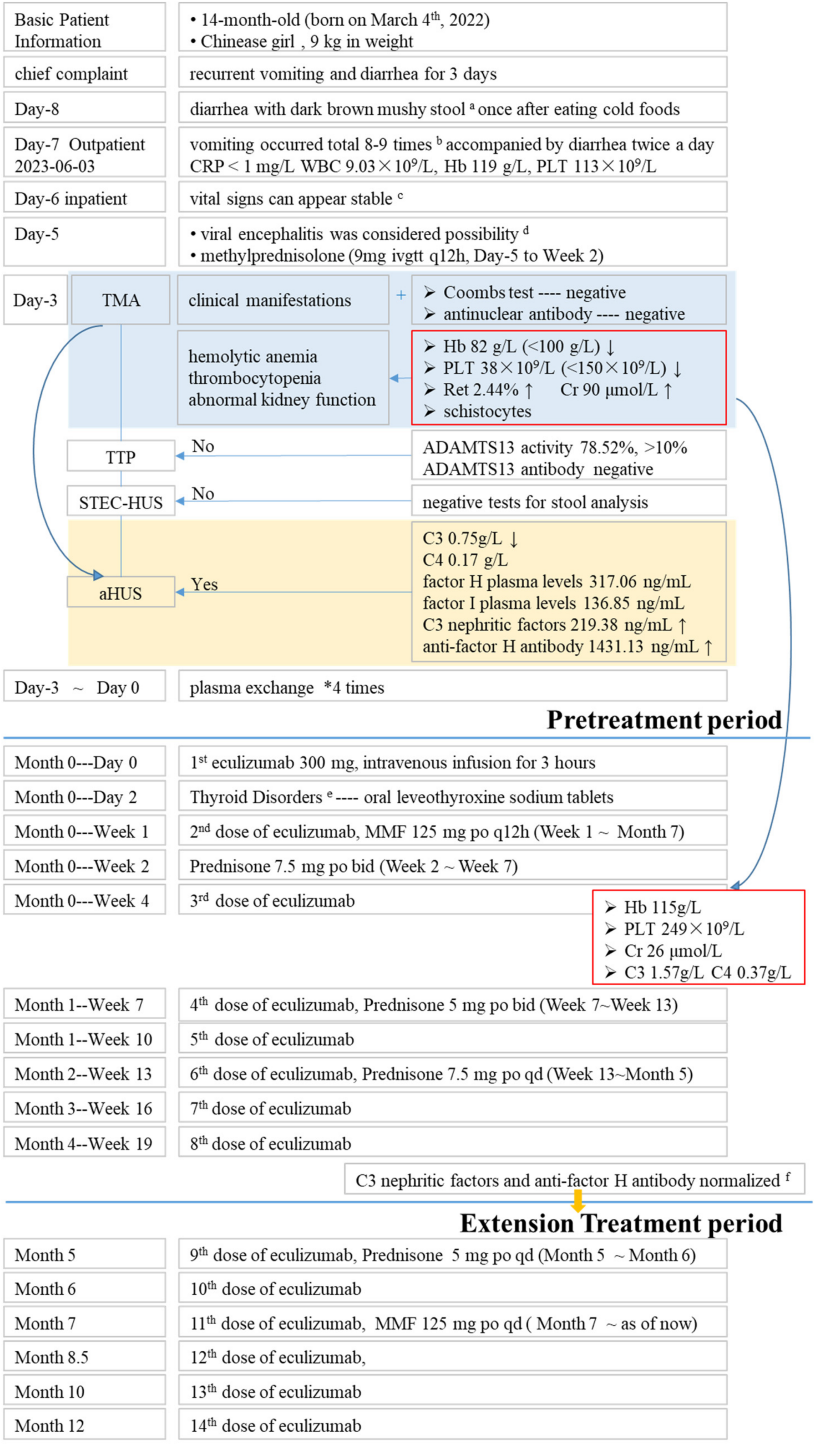
Brief description of treatment period. ^a^No foul odor, blood, or pus, and was not associated with significant abdominal pain or bloating. ^b^Initially, the vomitus consisted of gastric contents, but gradually changed to clear water-like fluid and eventually became dark green in color. The vomiting was accompanied by diarrhea, with 2 bowel movements per day. ^c^77 cm in height 23 times/min in breathing, 106 times/min in pulse, body temperature 36.2°C blood pressure 117/85 mmHg. ^d^Anti-infection, blood products and other symptomatic support treatments. ^e^FT3, 0.49 pmol/L FT4, 9.1 pmol/L TSH, 0.336 uIU/ml. ^f^C3 nephritic factors 114.06 ng/ml anti-factor H antibody 612.6 ng/ml.

**Figure 2:**
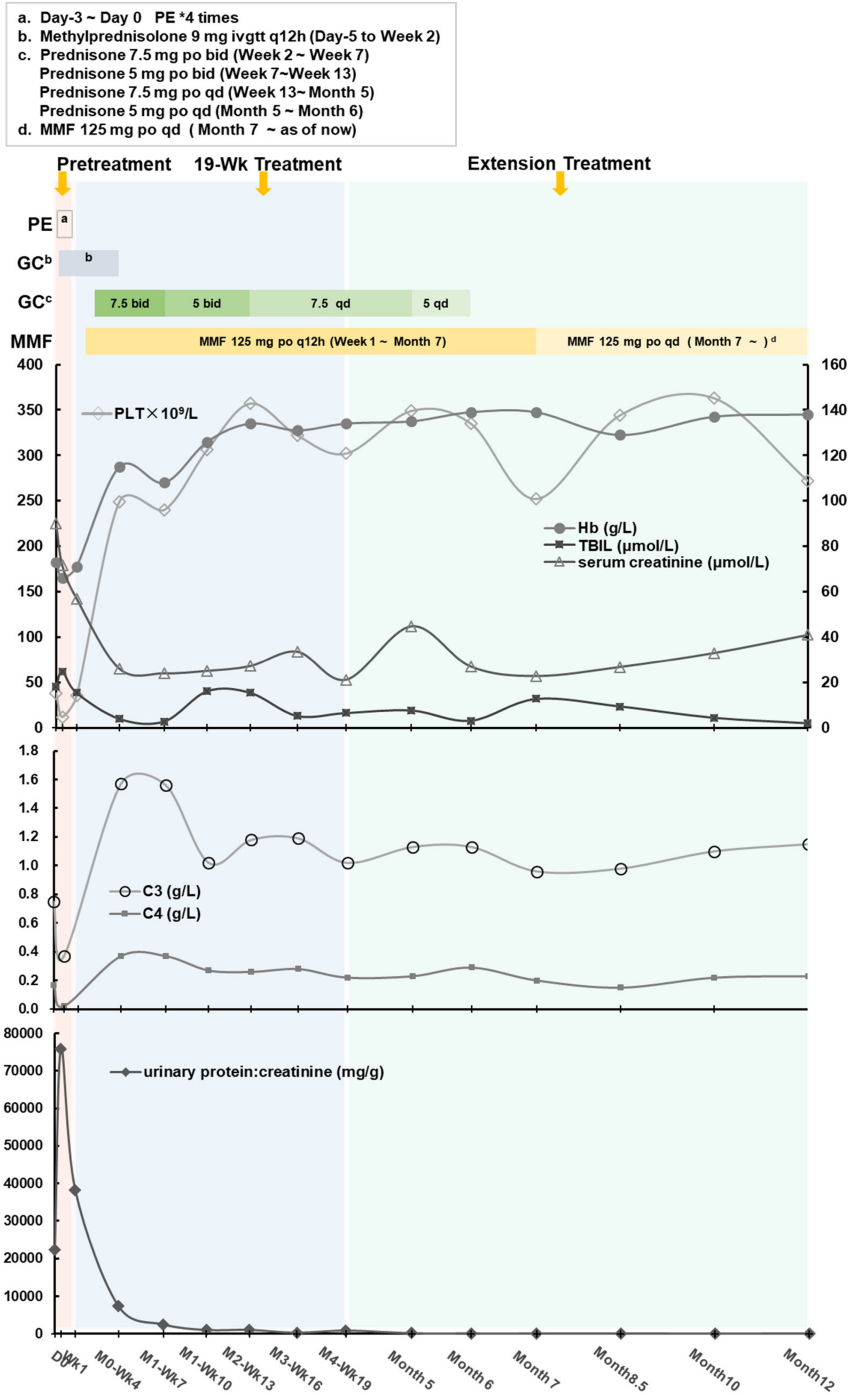
The individualized treatment regimen and the variations in clinical indicators across three periods.

The activity of A disintegrin and metalloproteinase with thrombospondin motifs 13 (ADAMTS13), a factor related to thrombotic thrombocytopenic purpura (TTP), was 78.52% (>10%, reference range: 42.16%–126.37%), and the patient tested negative for ADAMTS13 antibodies, ruling out TTP. No Shiga toxin-producing Escherichia coli, Salmonella, or enterohemorrhagic Escherichia coli O157 was detected, and the stool characteristics did not fit the presentation of Shiga toxin-producing E. coli hemolytic uremic syndrome (STEC-HUS). The complement C3 level was decreased at 0.75 g/L (reference range: 0.9–1.8 g/L), while C4 was normal at 0.17 g/L (reference range: 0.1–0.4 g/L). The patient had normal factor H plasma levels (317.06 ng/ml, normal range 246.60–417.69 ng/ml) and normal factor I plasma levels (136.85 ng/ml 12.26–333.02 ng/ml), elevated C3 nephritic factors (an autoantibody to the alternative pathway C3 convertase 219.38 ng/ml, normal range 40.25–210.36 ng/ml) and elevated anti-human complement factor H antibody levels at 1,431.13 ng/ml (reference range: 474.38–1,346.75 ng/ml). These findings, along with the absence of STEC infection, TTP, and secondary TMA, were consistent with a diagnosis of aHUS (Figures 1, 2). Plasma exchange (PE) was immediately initiated as treatment. After 4 times of PE, the patient still had thrombocytopenia, microangiopathic hemolytic anemia, and acute renal failure, with no significant improvement in relevant biochemical markers. The patient's condition remained critical, and the response to PE was not evident (Tables 1, 2).

**Table 1:** Pharmaceutical care in patient medical records.

Patient details	14-month-girl 9 kg in weight 77 cm in height 23 times/min in breathing, 106 times/min in pulse, body temperature 36.2℃ blood pressure 117/85 mmHg	
Chief complaint	Recurrent vomiting and diarrhea for 3 days	
Diagnosis	TMA-aHUS	
Results	Hb 82 g/L, PLT 38 × 10^9^/L, Ret 2.44%, Cr 90 μmol/L	
	ADAMTS13 activity 78.52%, ADAMTS13 antibody negative negative tests for stool analysis	
	C3 0.75 g/L, C4 0.17 g/L, factor H 317.06 ng/ml, factor I 136.85 ng/ml increased C3 nephritic factors and anti-factor H antibody	
Medication Safety Adverse drug reactions	Infection	(1) Completed vaccination according to the national immunization program (2) No antibiotic prophylaxis was given during the treatment period.
	Infusion reaction	No pre-treatment with antihistamines or acetaminophen or nonsteroidal antiinflammatory drug was given before each dose, and the patient did not experience infusion reactions.
	Thyroid disorders	The abnormal thyroid function on Day 2 could be related to the use of glucocorticoids or Eculizumab, as adverse drug reactions cannot be ruled out. Thyroid dysfunction improved with symptomatic treatment
Individualized dosing regimen	MMF 125 mg orally every 12 h (approximately548 mg/m^2^ per day) was added on day 10 based on high anti-factor H antibody 1,431.13 ng/ml, PLT (11 × 10^9^/L), and UACR (38,906.68 mg/g).	
Attempted dose reduction and discontinuation of eculizumab	The dosing interval was extended from every 3 weeks to once a month based on recover for anti-human complement factor H antibody and C3 nephritic factors. Discontinuation of eculizumab in aHUS patients is considered a justifiable and safe approach based on stable Cr, anti-factor H antibodies and absence of rare variants in complement genes.	

**Table 2:** Summary of Key laboratory test results throughout the treatment period.

	PLT (×10^9^/L)	Hb (g/L)	TBIL (μmol/L)	Cr (μmol/L)	UACR (mg/g)	urine protein (mg/dl)^a^	RBC (HP)^a^	C3 (g/L)	C4 (g/L)	CH50 (U/ml)	Coombs’ test	Factor H (ng/ml)	Factor I (ng/ml)	factor H antibody (ng/ml)	C3 nephritic factors (ng/ml)	Ret (%)	LDH (U/L)
Day -5	46	96	30.1	96.6		500	70–80	0.75	0.17	/	Negative					2.07	11
Day -4	38	49	28.1	85.6	28,075.75	150	50–60	/	/	/						2.22	/
Day -3	38	73	18.1	90	22,272.64	150	15–20	/	/	/		317.06	136.85	1,431.13	219.38	2.44	/
Day -2	40	60	27.1	78.7	37,858.25	500	40–50	0.41	0.09	29		/	/	/	/	/	/
Day -1	38	135	/	/	/	500	55–60	0.5	0.11	/			/	/	/	/	/
Day0	12	66	24.8	71.6	113,301.3	500	Full	0.37	0.02	9			/	/	/	16.74	1,425
Wk1	35	71	15.4	57	38,191.73	150	12–15	/	/	/			/	/	/	12.57	/
M0-Wk4	249	115	3.9	26	7,425.04	500	45–50	1.57	0.37	/			/	/	/	1.86	450
M1-Wk7	240	108	2.8	24	2,509.4	75	4–6	1.56	0.37	/			Normal	992.75	253.62	2.11	328
M1-Wk10	306	126	16.1	25.1	1,026.53	25	8–10	1.02	0.27	/			/	/	/	/	/
M2-Wk13	357	134	15.4	27.2	1,059.21	25	15–20	1.18	0.26	/			/	/	/	/	/
M3-Wk16	322	131	5.2	33.6	302.22	25	1–2	1.19	0.28	/			/	/	/	/	/
M4-Wk19	302	134	6.5	21.2	873.71	25	2–3	1.02	0.22	/			Normal	612.58	114.06	/	/
Month 5	349	135	7.6	44.8	161.67	Negative	2–3	1.13	0.23	/			/	/	/	/	/
Month 6	335	139	3.1	27	124.3	Negative	2–3	1.13	0.29	/			/	/	/	/	/
Month 7	252	139	12.7	22.8	48.17	Negative	None	0.96	0.2	/			/	/	/	/	/
Month8.5	344	129	9.4	26.8	41.32	Negative	None	0.98	0.15	/			/	/	/	/	/
Month10	363	137	4.4	33	85.43	Negative	None	1.1	0.22	<10			/	/	/	/	/
Month12	272	138	2	41	39.91	Negative	None	1.15	0.23	<10			/	/	/	/	/

^a^ Routine urinalysis.

After confirming that the child had completed vaccination according to the national immunization program and ruling out contraindications, on day 0 (2023-06-10), the first dose of 300 mg of Eculizumab was administered intravenously over a 3-hour period. (The subsequent doses 2nd∼11th were administered at the same dosage and duration.) During these 3 h period, vital signs of this pediatric patient was stable without any adverse reactions such as headache, tremor, hypertension, hypotension, cough, dyspnea, rash, diarrhea, nausea vomiting and etc. Two days (day 2) after medication, the levels of free triiodothyronine (FT3), free thyroxine (FT4), and thyroid-stimulating hormone (TSH) were found to be low (FT3: 0.49 pmol/L, FT4: 9.1 pmol/L, TSH: 0.336 uIU/ml). Symptomatic treatment with oral sodium levothyroxine tablets was initiated. Subsequently, the 2nd and 3rd dose of eculizumab were given at Week 1 and Week 4 respectively following the drug instructions and recommended guidelines. After then, hemoglobin (115 g/L), platelet count (249 × 10^9^/L), blood creatinine (26 μmol/L), C3 (1.57 g/L), and C4 (0.37 g/L) levels all returned to normal ranges, indicating significant improvement in the patient's condition. However, the Urine Albumin-to-Creatinine Ratio (UACR) remained high (7,425.04 mg/g), and therefore, losartan tablets (25 mg po qd) were administered to reduce urinary protein.

Eculizumab was administered at a dosage of 300 mg every 3 weeks (Week 7, 10, 13, 16, 19). After 8 doses, the levels of anti-human complement factor H antibody (612.6 ng/ml, normal range 474.38–1,346.75 ng/ml) and C3 nephritic factors (114.06 ng/ml, normal range 40.25–210.36 ng/ml) significantly improved, leading to an overall assessment of the patient's recovery. Consequently, the dosing interval was extended from every 3 weeks to once a month. Whole exome sequencing was performed on the patient, but no complement-related gene mutations were detected. As of now, with a disease course of over 8 months, the treatment regimen consists of a combination of Eculizumab, glucocorticoids (prednisone tablets), and mycophenolate mofetil (MMF), without prophylaxis antibiotics throughout treatment.

At present, the general condition of the patient is stable, and regular monitoring of TMA (hemoglobin, platelet count, blood creatinine, C3, and C4) every 3∼4 weeks shows values within the normal range. No evidence of TMA was found and aHUS did not recur neither.

## 3 Adverse drug reactions and management

### 3.1 Infection

The mechanism of action of Eculizumab involves high-affinity binding to the complement protein C5, inhibiting the cleavage of C5 into C5a and C5b. This prevents the formation of the terminal complement complex C5b-9, which plays a key effector role in extracellular killing capsule-forming bacteria, including *Neisseria meningitidis*, and the use of this medication may increase susceptibility to infection by encapsulated bacteria (11–15). It is recommend in drug prescribing information that meningococcal vaccination is required at least 2 weeks prior to the initiation of eculizumab therapy, or the administration of antibacterial drug prophylaxis for the first 2 weeks after vaccination if eculizumab treatment needs to start immediately. Additionally, children under 18 years old must be immunized against Haemophilus influenzae and Streptococcus pneumoniae infections, following the national immunization recommendations specific to each age group. It has also been suggested in the “Kidney Disease: Improving Global Outcomes” (KDIGO) conclusions (16) that antimicrobial prophylaxis should cover the entire period of Eculizumab treatment, until 2–3 months after discontinuation of Eculizumab.

(1) Prior to medication administration, the patient's family was carefully interviewed, and vaccination records were checked. The patient received meningococcal vaccine (Wuhan Biotech) on September 16th 2022, and Japanese encephalitis vaccine (Yuxi Watson) on January 5th 2023. Additionally, routine vaccinations including the 13-valent pneumococcal vaccine, polio vaccine, diphtheria-tetanus-pertussis vaccine, measles-mumps-rubella vaccine, Haemophilus influenzae type b vaccine, varicella vaccine, and enterovirus 71 vaccine have all been administered on time and are within their validity period.
(2) No antibiotic prophylaxis was given during the treatment period. The patient's family was specifically instructed to maintain personal hygiene, avoid cross-infections. In case of symptoms such as cough, sputum production, abdominal pain, diarrhea, or headache, immediate medical attention should be sought.
(3) So far, after the administration of 11 doses of eculizumab, no adverse drug reactions, such as Neisseria meningitidis infection, have been observed.

### 3.2 Infusion reaction

Eculizumab is a recombinant humanized monoclonal antibody with high homology to humans and low immunogenicity. No pre-treatment with antihistamines or acetaminophen or nonsteroidal antiinflammatory drug was given before each dose, and the patient did not experience infusion reactions such as fever, rash, diarrhea, or vomiting during or after the administration of the 11 doses.

### 3.3 Thyroid disorders

Prior to initiation of eculizumab treatment at Day-5, thyroid function tests showed that FT3 2.75 pmol/L, FT4 12.97 pmol/L, TSH 0.459 uIU/ml. No special treatment was given at that time. On the Day2 (2 days after the first dose), FT3 levels decreased to 0.49 pmol/L, FT4 levels decreased to 9.1 pmol/L, and TSH levels decreased to 0.336 uIU/ml. Possible reasons for this decrease are as follows:

(1) There are no reported adverse reactions causing thyroid function abnormalities related to aHUS or Eculizumab in literature. However, due to the low incidence rate and limited number of cases using Eculizumab, adverse drug reactions cannot be completely ruled out. (2) Medications that can affect thyroid function include (17) iodine, lithium, tyrosine kinase inhibitors, immune checkpoint inhibitors, interferon-alpha and interleukin-2, amiodarone, and glucocorticoids (prednisone >20 mg/day), among others.

Since the patient started receiving methylprednisolone at a dose of 18 mg/day (equivalent to prednisone 22.5 mg) on Day -5 of hospitalization, and thyroid function abnormalities occurred 8 days later (Day 2), additional treatment with levothyroxine sodium tablets at a dose of 37.5 μg once daily was initiated, leading to improvement. At the 4th dose of Eculizumab, T3 levels were 0.94 pmol/L, and TSH levels were 0.778 uIU/ml. Levothyroxine sodium tablets were discontinued, and at the 5th dose, T3 levels were 7.14 pmol/L, and TSH levels were 1.624 uIU/ml, showing no significant abnormalities. Based on comprehensive analysis, the abnormal thyroid function on Day 2 could be related to the use of glucocorticoids or Eculizumab, as adverse drug reactions cannot be ruled out.

## 4 Discussion

### 4.1 Prevention and treatment of adverse drug reactions

(1) In this case, the patient had received complete immunization prior to the onset of illness and did not receive antibiotic prophylaxis during medication. There were no occurrences of meningococcal or other infections during the 11 doses of eculizumab. It has been reported (11) that, prophylactic oral administration of amoxicillin may be effective for preventing meningococcal infection under treatment with eculizumab in infants aged < 9 months who are not vaccinated with meningococcal vaccine. (2) For this pediatric patient, no premedicate including antihistamine or acetaminophen or other nonsteroidal antiinflammatory drug is given prior to each infusion. Eculizumab -induced infusion reactions such as fever, rash, diarrhea, vomiting was not observed during treatment. (3) Except for thyroid dysfunction after the first two doses, which improved with symptomatic treatment, no other adverse drug reactions occurred.

### 4.2 Development of an individualized dosing regimen

This patient was born on March 4th 2022, and only 14-month-old, the onset of TMA (aHUS) occurred on June 2nd 2023, with rapid progression and critical condition. The diagnosis of aHUS was made on the 3rd day (day -3) after hospital admission. After 4 times of plasma exchange and immunosuppressive therapy, the patient's response was inadequate. On day 0, eculizumab treatment was initiated with a single dose of 300 mg administered intravenously over 3 h. According to the KDIGO 2017 conclusions (16) and reference (18), if anti-complement factor H antibodies are positive, concomitant administration of glucocorticoids and immunosuppressive agents (cyclophosphamide, rituximab, or mycophenolate mofetil) is recommended during eculizumab treatment to effectively suppress the production of anti-factor H antibodies, reduce antibody titers, inhibit excessive tissue inflammation, and improve the prognosis of the patient. After receiving glucocorticoids (methylprednisolone 9 mg ivgtt q12h, from Day -5 to week 2) and two doses of eculizumab (week 0 and week 1), the patient has sustained high levels of anti-factor H antibodies (1,431.13 ng/ml), inadequate recovery of platelet count(11 × 10^9^/L), and elevated urinary protein levels (UACR 38,906.68 mg/g). Considering the benefits and risks of medication, we have made the decision to incorporate mycophenolate mofetil (MMF) 125 mg orally every 12 h (approximately 548 mg/m^2^ per day) on day 10.

### 4.3 Attempted dose reduction and discontinuation of eculizumab

The duration of eculizumab treatment and the appropriate time for discontinuation are still controversial. So far, there is no evidence to support lifelong therapy in all aHUS patients. The KDIGO 2017 conclusions (16) recommend that discontinuation can be considered on a case-by-case basis in patients after at least 6 to 12 months of treatment and at least 3 months of normalization (or stabilization in the case of residual chronic kidney disease) of kidney function. Several studies have provided evidence on the safety of discontinuing C5 receptor blockade in patients without detectable pathogenic rare complement variants. (1) For instance, a study (19) reported 2 clinical trials involving patients with aHUS over 12 years of age, who received eculizumab for 26 weeks and during long-term extension phases. The median duration of eculizumab treatment was 64 weeks and 62 weeks, respectively. These all patients received meningococcal vaccination at least 14 days before the initiation eculizumab treatment or they received prophylactic antibiotic therapy until 2 weeks after vaccination. (2) The study (10) observed the discontinuation of eculizumab in fifty-five patients (including 19 children) with a mean treatment duration of 16.5 months. In individuals with aHUS, the risk of aHUS relapse following eculizumab discontinuation is primarily associated with the presence or absence of a rare variant in a complement gene, both in children and adults. A strategy of eculizumab discontinuation in aHUS patients based on complement genetics is deemed reasonable and safe. This approach enhances the management and quality of life for a considerable proportion of aHUS patients while also reducing treatment costs. (3) The administration of anti-C5 treatment is typically continued for a minimum period of 3–6 months following the acute phase of aHUS and until the serum creatinine levels have stabilized (20). Lifelong treatment with a C5 inhibitor is no longer universally applicable in the management of all aHUS patients. Multiple studies (10, 21–24) have suggested that discontinuation of C5 blockade is safe for patients lacking detectable pathogenic rare complement variants. (4) The information provided in another reference (11) describes a case report involving two infants who are cousins and were diagnosed with aHUS at the age of 4–5 months. Eculizumab therapy was administered to these infants for a duration exceeding 3 years and 1 year, respectively, as a treatment for aHUS. (5) Furthermore, there is a systematic review (25) published in Pediatrics Nephrology in January 2023. It highlights the lack of consensus regarding the optimal duration and cessation of eculizumab therapy, emphasizing the need for further clinical evidence from application cases. Currently, there are no standardized guidelines for the dosing intervals or treatment duration of eculizumab. The individualized treatment regimen, including adjustments to dosing intervals, is based on the patient's underlying condition, response to the medication, and adverse reactions. The treatment plan for this patient is a single case and is shared as a reference for possible adjustments. It is important to note that the treatment regimen for another pediatric patient is not exactly the same, which will be part of our next work.

To summarize, discontinuation of eculizumab in aHUS patients who exhibit stable serum creatinine and anti-factor H antibodies, also do not possess rare variants in complement genes is considered a justifiable and safe approach. For this pediatric patient, eculizumab was administered according to the drug prescribing information for a total of 8 doses. Afterward, the administration interval was increased from once every 3 weeks to once per month starting from the 9th dose. To date, there have been no instances of relapse.

## 5 How the experience in this case report will affect clinical practice

### 5.1 Personalized treatment plans

This case highlights the importance of tailoring the dosing and treatment schedule of eculizumab based on individual patient characteristics and response markers. This approach can optimize therapeutic outcomes and minimize potential side effects.

### 5.2 Monitoring and adjusting treatment

The case demonstrates the necessity of regular monitoring of complement activity and antibody levels to guide treatment adjustments. This can serve as a reference for other clinicians managing similar cases.

### 5.3 Safety and efficacy

The positive outcome without significant adverse reactions supports the safe use of eculizumab in pediatric patients with aHUS, providing reassurance to clinicians about its efficacy and safety profile in young children.

### 5.4 Future practice

If similar situations are encountered again, we would continue to employ a personalized approach, with careful monitoring and dose adjustments based on clinical and laboratory findings. This case will inform future practices by providing a detailed protocol for managing complex cases of aHUS with eculizumab.

In conclusion, while the relevance to other cases may be limited, the detailed insights gained from this case can contribute to refining treatment strategies for aHUS, ensuring better patient outcomes in similar future cases.

## 6 Patient perspective

At the onset of the child's symptoms such as diarrhea and vomiting, they were initially perceived as common gastrointestinal issues. However, the rapid deterioration of the condition plunged us into a crisis, with the child's life hanging in the balance. As we actively collaborated with the medical team, it became evident that traditional treatment approaches seemed ineffective, causing heightened anxiety. As our child is an *in vitro* fertilization baby, we cherish her dearly, and thus we readily agreed to the use of Eculizumab, despite its high cost.

The journey of treating a child with a rare disease was far from smooth, especially considering the limited experience with drug therapies. Nevertheless, as parents, we were determined to work hand in hand with the doctors and pharmacists to explore this novel treatment avenue. The doctors informed us about the potential risk of meningococcal infection associated with eculizumab, prompting us to review the child's vaccination records, confirming compliance with the national immunization program. Subsequently, after thorough discussions with the medical team, we commenced the treatment. Throughout the entire treatment process, we strictly adhered to the advice of the doctors and pharmacists, closely monitoring any signs of allergic reactions or other related adverse effects. Fortunately, the child did not experience any drug-related serious adverse reactions, even in the absence of prophylactic antimicrobial drugs or other adjunctive medications.

Following the 11 doses of eculizumab therapy, there was a noticeable improvement in the child's condition, bringing immense relief to us. Subsequently, we maintained regular visits to the hospital for monitoring various parameters to ensure the child's continued health stability. While occasional instances of diarrhea and respiratory infections occurred during the treatment, prompt medical attention enabled effective management. Presently, the child has not only triumphed over the illness but has also regained their health, allowing her to enjoy life, play, and learn like any other child.

The doctor informed us that after the acute phase was under control, the child could be as healthy and lively as they were before falling ill. Indeed, this has proven to be the case. This experience has deeply underscored the value of life, and we are grateful for the medical team's treatment and care. May our journey serve as a beacon of hope and courage for other families, enabling more children to thrive and grow amidst love.

## 7 Follow-up and current status

At the time of submission, the patient had received 11 doses of eculizumab. Since then, the patient has received an additional 3 doses, bringing the total to 14 doses by this month. The dosing interval has gradually been extended from once a month to once every 1.5 months, and now we are trying once every 2 months. Glucocorticoids were tapered off in Month 6 (December 2023), and MMF was reduced to 125 mg po qd in January 2024 (Month 7). The patient's condition is currently stable, with no adverse drug reactions reported.

Due to the exploratory nature and many uncertainties, the parents have been informed to report any abnormal situations to the physician promptly and to visit the hospital for follow-up if necessary. To date, the disease under control and stability without relapse during the treatment period; symptoms improved as parents said there was no difference with other children in daily life; indicators (blood routine tests, blood biochemistry, complement, urine routine tests) were normal and stable (Figures 1, 2; Table 2). After 12 months of treatment, no infections or allergic reactions have been observed.

## 8 In conclusion

We experienced and successfully treated a rare case of aHUS in a 14-month-old little girl. After confirming the diagnosis, plasma exchange was performed for 4 times with inadequate response, and subsequently, treatment with eculizumab was initiated. Before medication, the patient had completed vaccination according to the national immunization program especially including meningococcal vaccine, and no prophylaxis antibiotics were administered during the medication period. There were no infusion reactions or meningococcal infections during the treatment. Except for thyroid dysfunction after the first two doses, which improved with symptomatic supportive treatment, no other adverse drug reactions occurred. The patient tested positive for anti-factor H antibodies. In addition to glucocorticoid treatment (methylprednisolone 9 mg IV every 12 h, from Day -5 to week 2) and two doses of eculizumab (week 0 and week 1), there were insufficient recovery of PLT and urine albumin-creatinine ratio, MMF 250 mg daily (approximately 548 mg/m^2^ per day) was added on Day 10 for combination therapy. Following the administration of the initial 8 planned doses and the observed enhancement in anti-complement factor H antibodies, the dosing frequency was adjusted from every 3 weeks to a monthly schedule commencing from the 9th dose. After receiving eculizumab treatment for approximately 8 months, this patient achieved a notably improved recovery from aHUS without experiencing any relapse thus far. Consequently, it is expected to discontinue the medication after 9∼10 months of treatment and then follow up.

However, the limited number of cases and the relatively brief duration of observation render it challenging to draw definitive conclusions. Furthermore, there is a scarcity of data on pediatric aHUS associated with anti-complement factor H antibodies in the eculizumab era, which makes it nearly impossible to effectively compare the benefit-to-risk ratios of different therapeutic strategies.

The successful diagnosis and treatment of this 14-month-old Chinese girl with aHUS offer valuable insights and serve as a basis and reference for the formulation of individualized treatment strategies for pediatric patient with aHUS.

## Data Availability

The original contributions presented in the study are included in the article, further inquiries can be directed to the corresponding authors.
